# Cognitive appraisal processes and health: from bench to bedside

**DOI:** 10.3389/fpubh.2026.1713379

**Published:** 2026-03-02

**Authors:** Carolyn E. Schwartz, Richard L. Skolasky, Bruce D. Rapkin, Katrina Borowiec, Joel A. Finkelstein

**Affiliations:** 1 DeltaQuest Foundation, Inc., Concord, MA, United States; 2 Department of Medicine and Orthopaedic Surgery, Tufts University Medical School, Boston, MA, United States; 3 Department of Orthopaedic Surgery and Physical Medicine and Rehabilitation, The Johns Hopkins University School of Medicine, Baltimore, MD, United States; 4 Department of Epidemiology and Population Health, Albert Einstein College of Medicine, Bronx, NY, United States; 5 Department of Measurement, Evaluation, Statistics, and Assessment, Boston College Lynch School of Education and Human Development, Chestnut Hill, MA, United States; 6 Department of Surgery, University of Toronto, Toronto, ON, Canada; 7 Division of Orthopedic Surgery, Sunnybrook Health Sciences Centre, Toronto, ON, Canada; 8 Division of Spine Surgery, Sunnybrook Health Sciences Centre, Toronto, ON, Canada

**Keywords:** cognitive appraisal processes, cognitive-behavioral therapy, intervention, response shift, review

## Abstract

**Background/objectives:**

After two decades of cognitive-appraisal research in spine surgery and other medically ill patients, the present work reviewed the published literature on cognitive-appraisal processes and health outcomes to determine key findings and considerations, and how they point to appraisal-assessment based interventions to improve clinical outcomes.

**Methods:**

The present work reviewed the published literature on cognitive-appraisal processes and health outcomes to determine key findings and considerations, and how they point to interventions to improve clinical outcomes. These implications were further examined in terms of when such appraisal processes should be addressed and how individual differences should be explicitly considered.

**Results:**

The literature review identified 53 articles, of which 26 were retained for further review and 27 were excluded because they either (1) used a different definition of cognitive appraisal or (2) focused solely on measurement development; methodological or statistical development; or theory development. The findings from the retained studies highlighted the following cognitive-appraisal processes in order of frequency of mention: Sampling of Experience, Standards of Comparison, Frame of Reference, and Combinatory Algorithm. Empirical findings also generally supported more interventions on appraisal processes after surgery rather than before. An appraisal-assessment based intervention is proposed that builds on the empirical evidence.

**Conclusion:**

It is our hope that the present work has provided the next logical step in response-shift research, moving from basic, foundational findings to implications for clinical interventions that can help medically ill patients recover in more lasting and deeper ways from surgery and other health state changes.

## Introduction

Translational research is critical for turning basic-science findings into clinical treatments, but such research is less common in behavioral science. Although behavioral science may test clinical interventions with rigorous clinical trials and real-world-evidence designs, it is uncommon for basic-science findings to have clear clinical implications that merit intervention development. In the field of quality-of-life (QOL) research, patient-reported outcome measures have been used as one-time screening questionnaires, as monitors of progress over time, and to enhance doctor-patient communication (1–3). Recent empirical findings that address QOL appraisal and response shift suggest a potential role of appraisal-assessment based intervention in clinical practice. Response shift refers to the idea that when individuals experience a health-state change (catalyst), they may change their internal standards, values or conceptualization of the target construct (1, 2). These response-shift effects, referred to as recalibration, reprioritization, and reconceptualization, respectively, can make it challenging to interpret changes in longitudinal data because they may serve to hide changes due to the effects of adaptation (1, 2).

Many studies of response shift rely on statistically complex approaches to operationalizing response-shift effects [e.g. (3–5)], which makes it more challenging to identify inroads for affecting change. In contrast, response-shift studies using more direct measures of the cognitive-appraisal processes seem to point more unambiguously to individual differences that may be amenable to behavioral interventions.

To further contextualize our current study, below we will provide an overview of the theoretical and methodological foundations for direct measures of cognitive-appraisal processes, as well as background information on cognitive-behavioral interventions for medical health outcomes.

### Theoretical foundations for direct measures of cognitive-appraisal processes

Studies that utilize direct measures of cognitive-appraisal processes consider appraisal from the framework of the psychology of survey response (6) rather than the Lazarus and Folkman (7) definition of primary and secondary appraisal focused on the individual’s characterization of a stressor as threatening or harmful. The former framework considers four aspects of how individuals think about QOL: how they define QOL and what goals are considered meaningful to them (Frame of Reference); what experiences they sample when they rate their QOL (Sampling of Experience); to whom they compare themselves (Standards of Comparison), and what aspects are emphasized when evaluating their QOL (Combinatory Algorithm) (2, 8–10).

**Figure 1:**
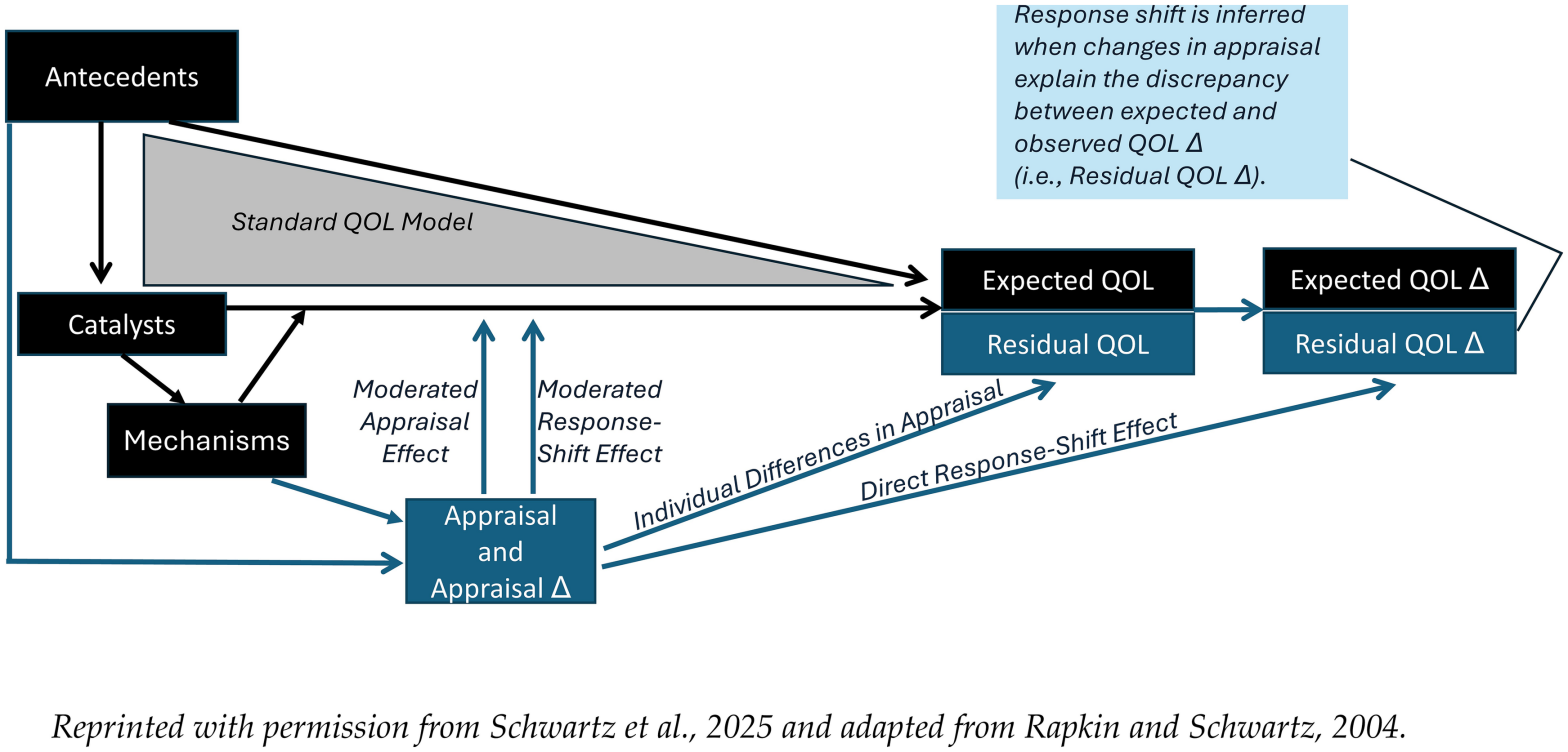
The Rapkin and Schwartz QOL appraisal model (2, 8). Reprinted with permission from Rapkin and Schwartz (2) and Schwartz et al. (11).

Over the past two decades, using these direct measures of cognitive-appraisal processes has paved a long empirical path beginning with the Rapkin and Schwartz Appraisal Model (2, 11) (Figure 1). This model hypothesizes that catalysts, such as changes in health, treatment, or life events, are directly related to QOL and QOL change (QOL Δ), and the effects of antecedents (e.g., demographic factors, personality, culture, and historical influences) on QOL are mediated through catalysts. Mechanisms encompass behavioral, cognitive, and affective processes to accommodate or cope with changes in catalysts, such as initiating social comparisons, reordering goals, spiritual practice and reframing expectations. It should be noted that expectations and appraisal processes are distinct both conceptually and operationally. Expectations reflect a hope or subjective belief about a situation prior to the situation (e.g., After the surgery, I will be able to run a marathon). In contrast, appraisal processes reflect how one thinks about or evaluates the situation in the moment and may also reflect tendencies for evaluating across situations (e.g., When I think about my surgery experience, I focus on the negative aspects I had to deal with). Although clearly related because both are cognitive processes, expectations can be set by the person’s healthcare provider when describing the medical situation. Appraisal processes may be set by a number of antecedent conditions and may be modified once one becomes aware of the tendency and sets an intention or goal to change to a different approach for evaluating life’s challenges. Because appraisal assessment focuses on individuals’ ways of thinking about their life in the moment, it can provide important content for self-reflection and evaluation in CBT. For example, an individual may state that they are only thinking about the impact of illness on others or only focusing on loss, without examining the basis for those appraisals. Aspects of appraisal that are absent may also be noteworthy, e.g., ignoring the possibility of regaining function or expanding social roles. Appraisal assessment can open the door to discussion of issues such as these, particularly as they pertain to an individual’s health-related QOL.

In addition to stable cognitive-appraisal processes impacting QOL and QOL Δ (direct and moderated effects due to individual differences in appraisal), this model hypothesizes that changes in appraisal may affect change in QOL ratings directly (“direct response shift” path) or by attenuating or amplifying the impact of catalysts (“moderated response shift” path).

### Methodological foundations for direct measures of cognitive-appraisal processes

The Rapkin and Schwartz Appraisal Model (2) served as the theoretical foundation for creating and testing a series of appraisal measures in over 6,000 patients with the aim of yielding a relatively brief measure of appraisal that was “portable” to a range of patient populations and research contexts (i.e., cross-sectional or longitudinal, interview format or web-based survey). Beginning with the QOL Appraisal Profile (QOLAP) version 1, which was comprised largely of open-ended questions (2), the work eventually yielded a series of closed-ended measures that were faster to quantitate (12, 13) and maintained the four domains of the Rapkin and Schwartz Appraisal Model (2). The current recommended measure, the QOLAP_v2_-Short Form (14), includes 28 suggested items reflecting the four cognitive-appraisal domains. From an original pool of 84 items (13) developed from mixed-methods research, these 28 items were selected on the basis of their ability to explain variance in demographic characteristics (14). Furthermore, this appraisal-based empirical trail developed variations of state-of-the-art longitudinal statistical analyses to hone the capture of the four aspects of response shift (11, 15, 16).

### The role of appraisal in health behavioral interventions

Assessment of what people are thinking or what matters to their QOL is not novel in clinical practice. It is intrinsic to good clinical practice. To date, the assessment of patients’ priorities and concerns has been largely dependent on the “soft” skills of the clinician as well patients’ predilection for introspection and communication. The systematic assessment of appraisal thus opens the door to a more consistent and rigorous approach for communicating about what and how patients are thinking about QOL. Once such communication is achieved, it can be used in conjunction with a wide range of educational and counseling intervention techniques aimed at modifying patients’ cognitions and behaviors. Supplementary Text 1 provides a brief review of the impact in medical populations of several prominent examples of “Cognitive and behavioral interventions” (CBT), including Dialectical Behavioral Therapy, Acceptance-based interventions, and Motivational Interviewing (MI). These CBT interventions are familiar to clinicians, as they intrinsically invoke appraisal and self-reflection.

### Purpose of the current study

As research on cognitive-appraisal processes expanded the range of patient populations and addressed more clinical rather than methodological considerations, the clinical implications of study findings became more prominent. It now seems like the appropriate time to develop an intervention based on these two decades of research to determine whether it is possible to help individuals to modify their cognitive-appraisal processes and to ultimately improve their health outcomes.

The present work thus sought to review the published literature on cognitive-appraisal processes and health outcomes from our group and other researchers to determine key findings and considerations, and how they point to interventions to improve clinical outcomes. For the sake of clarity regarding the catalyst of interest, we will focus the proposed intervention on the clear catalyst of spinal surgery. This catalyst was selected because its timing can be controlled when designing an intervention in contrast to the diagnosis of a chronic or life-threatening condition.

## Methods

This project follows a narrative or scoping review framework and is not intended to be a systematic review. We began by searching the literature using the OVID and Google Scholar platforms to find published papers addressing cognitive-appraisal processes and health outcomes. We focused on these two search platforms because several scoping reviews done by members of our group found that including other platforms resulted in less relevant and more duplicate papers, and that these two platforms complemented each other well without yielding irrelevant or duplicate papers.

Eligible papers were published in English and directly addressed the same definition of cognitive appraisal processes used in the context of response-shift research (2, 8), i.e., building on the psychology of survey response (6). Papers were deemed ineligible if they used a different definition of appraisal, or focused on theory only, measurement development only, or statistical methodological development only.

Included papers were then evaluated by the lead author (CES) and these results were reviewed by two other co-authors (KB and RLS) to abstract the key empirical findings linking cognitive-appraisal processes to health outcomes, to record the specific cognitive-appraisal processes concerned, and to note the clinical implications of the findings with an eye toward codifying an appraisal-based clinical intervention. Study abstraction was done sequentially and any disagreements among reviewers were discussed and resolved.

These implications were further examined in terms of when such appraisal processes should be addressed (i.e., pre- or post-surgery) and how individual differences should be explicitly considered. The end goal was to develop an intervention that could be tested in a randomized controlled trial to evaluate the intervention’s impact.

## Results

**Figure 2:**
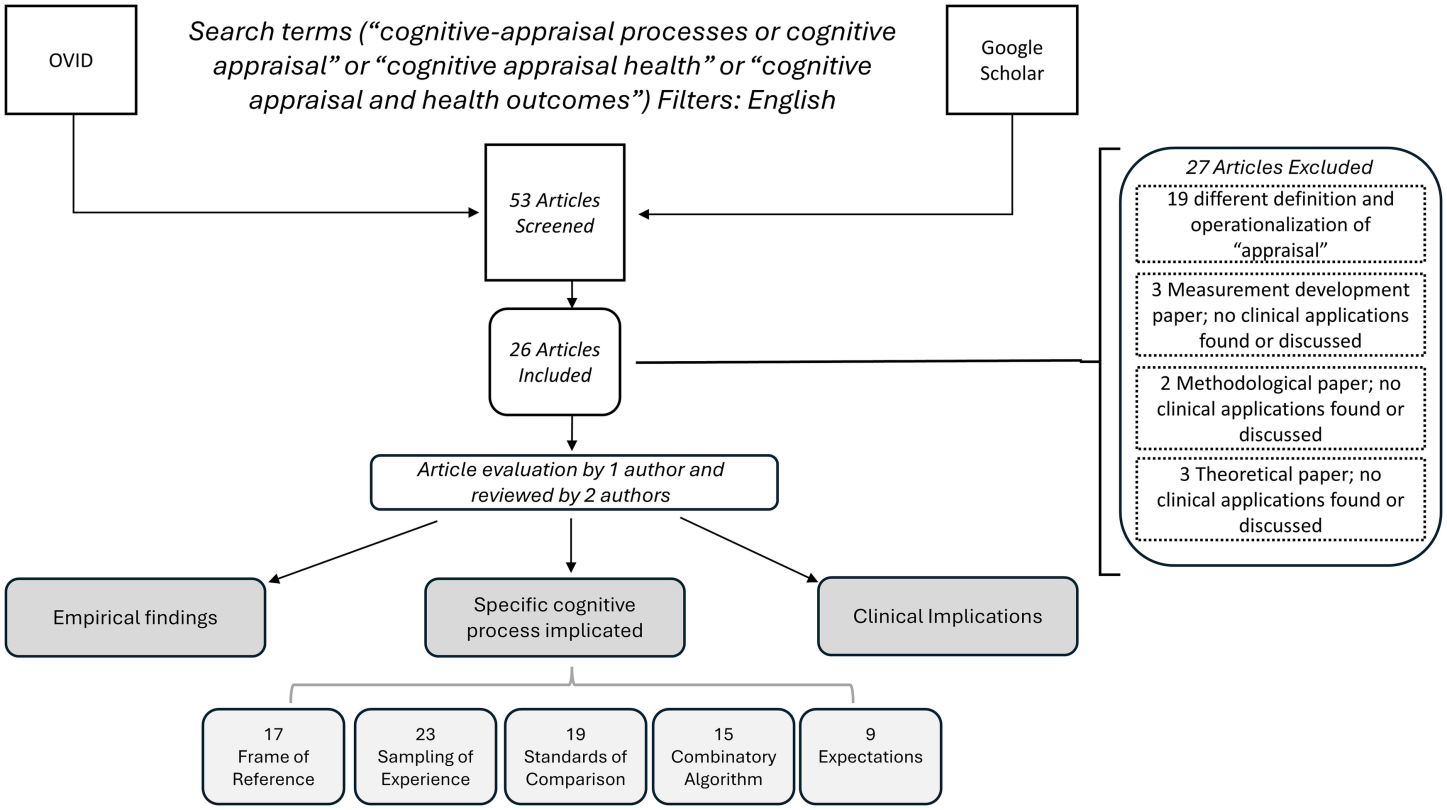
Flow chart of article selection process.

The literature review identified 53 articles, of which 26 were retained for further review (11, 17–41). Twenty-seven papers were excluded (2, 8–10, 12–14, 42–61) because they used a different definition of cognitive appraisal (i.e., the Folkman and Lazarus definition) (42–55, 57–61), focused solely on measurement development (12–14), focused solely on methodological or statistical development (8, 56), or focused solely on theory development (2, 9, 10). Figure 2 provides a flow chart of the article-selection process. Appendix I provides the summary for the included papers, and Supplemental Table 1 provides a listing of the excluded papers and reasons for exclusion.

Of the retained papers, the study results highlighted the following cognitive-appraisal processes in order of frequency of mention: Sampling of Experience (23), Standards of Comparison (19), Frame of Reference (17), and Combinatory Algorithm (15). The importance of treatment-related expectations was also noted in 9 papers and thus deemed worthy of attention in an intervention. Of note, expectations is considered in the Rapkin and Schwartz Appraisal Model as an antecedent (i.e., stable characteristics of an individual prior to the health state change).

**Figure 3:**
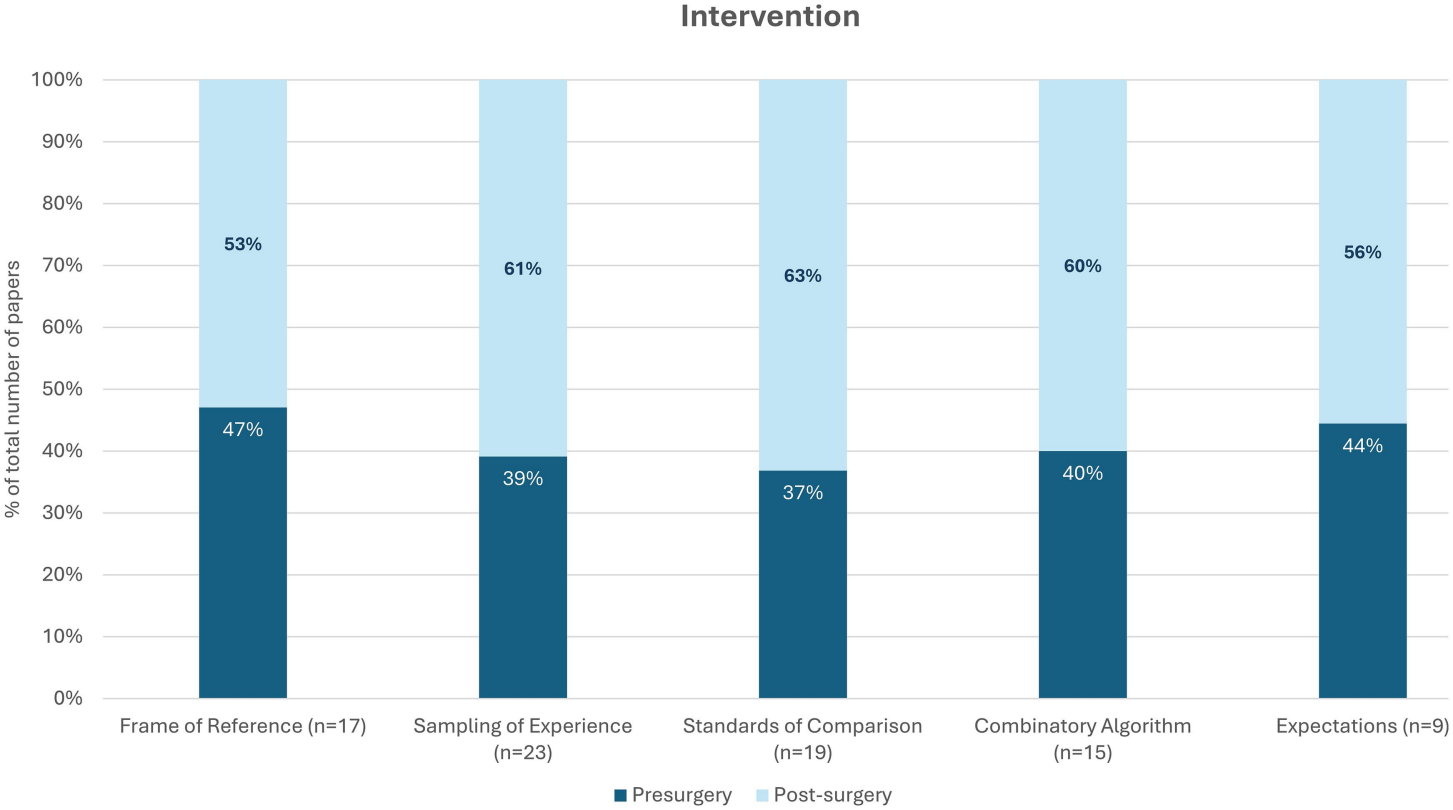
Empirical recommendations regarding timing of appraisal intervention.

As our intervention is applied to spinal disorders, we then focused on the timing of the clinical implications to clarify which aspects of appraisal should be addressed before surgery and which after surgery. While all appraisal processes were implicated both before and after surgery, the empirical findings generally supported more intervention on appraisal processes after surgery rather than before (Figure 3). This may be due to an unavoidable artifact in research that focuses on outcomes over time after the catalyst (i.e., post-surgery).

Finally, we noted findings from specific papers that highlighted subpopulations that should be addressed in a distinct manner. This focus on individual differences is consistent with appraisal theory (2, 8, 9) but has only been addressed in studies published in 2024 and later (25, 28, 38), perhaps due in part to advances in statistical methods as well as the availability of larger data sets to test subpopulation hypotheses. These papers highlighted distinct cognitive-appraisal foci for individuals with long-standing disability (28), with low patient activation (i.e., sense of agency in their healthcare) (38), at risk of opioid use disorder (40), for those who are low in social support, facing greater financial challenges (27), and for those dealing with long-term depressive symptoms (25).

### The appraisal intervention

QOL Appraisal Assessment can help individuals to better articulate, unpack, and reflect upon complex factors that bear on the sense of well-being and adaptation to illness. QOL Appraisal Assessment may best be used in the broader context of patient counseling and support. It is not intended to be a “stand-alone” approach to psychosocial intervention. Rather, appraisal assessment is intended to guide patients and providers by illuminating key considerations that enter into an individual’s QOL. Accordingly, the emerging appraisal-based intervention might inform the content of CBT-related interventions, rather than being a wholly new intervention. In other words, an appraisal-based intervention might use similar or the same techniques as a CBT intervention but would focus more explicitly on specific content that we have found to be meaningfully associated with better health outcomes over the course of two decades of research.

The content of the emerging appraisal-assessment based intervention is shown in Table 1, with similar topics in pre- and post-surgery intervention periods shown in similar colored shading. This table provides a general overview of the intervention, while Table 2 focuses on components designed for specific subpopulations. Both periods of the intervention use the QOLAP as a communication tool to help characterize and communicate the individual’s current concerns and thinking. This assessment tool can also provide valuable feedback on change over time.

**Table 1:** Emerging intervention to address cognitive-appraisal processes.

**Topics**	**Content**
**Pre-Surgery**	
QOLAP as communication tool	Use the QOLAP to help identify the individual's current concerns so that conflicts can be anticipated and avoided. Use it as an opportunity to think through the ramifications of treatment in terms of the individual's goals and activities
Encouraging patient activation and education (prehabilitation)	Before surgery, explain the normal course of recovery and the effects of surgery. Develop a plan for rehabilitation that includes family members, broken down in smaller steps by week since surgery
	Preoperative education might focus on improving patient knowledge, their feeling prepared, reducing negative thinking, and increasing levels of physical activity after spine surgery. If someone is overly focused on the seriousness of their situation, it may mean that additional problems are present (i.e., things really are more serious compared to most patients) or that an individual’s expectations are off (e.g., they expect a recovery that is faster than realistic). Directly addressing the individual’s context and their expectations could be done with a “prehabilitation” type approach
	A prehabilitation approach might involve talking with patients about how focusing on preparing one's family for one's health changes is not adaptive (i.e., associated with worse outcomes), and that a better approach would be to focus on improving their living situation and on activities that help them maintain a positive outlook). Since this association may also reflect on the patient’s family situation, prehabilitation might also include consultations and education for caregivers, to help them better understand what to expect following surgery. The clinical implications of this work may involve practical support for life challenges, and emotional support to reframe dependency during recovery so that it is experienced as less worrisome
	Early on (at pre-surgery), encourage patients to expect to do exercise and other recreational activities after surgery
Address mental health	Mental health should be explicitly addressed in all sessions because poor mental health colors the patient's focus after surgery to be more negative
	Prior to surgery, address depressive symptoms Some appraisal processes may be helpful and adaptive at one time and not another. For example, comparing one's self to others in multiple ways seems advantageous prior to surgery but not after, for some depressed patients but not others (i.e., those whose depression did not improve)
Promoting adaptive cognitive-appraisal processes	Encourage focusing on recent events, and not to focus on how others see them
	For those low in social support, appraisal processes characterized by emphasizing the negative were particularly deleterious. For those with greater financial challenges, appraisal processes focused on obligations and getting used to changes were protective
	For individuals with long-standing disabling conditions, the intervention might focus partituclarly on improving their social-support network more than changing appraisal processes. These patients might seek to create contexts that are more similar to work environments with similar benefits: regular social contact, a meaningful role that is valued, and some kind of reward or compensation for regular and sustained participation
	If an individual is using opioids daily before surgery, they are at high risk of OUD. For these individuals, interventions might emphasize the adoption of more problem-resolution goals at pre-surgery. The lagged effect of baseline goals points to the importance of early intervention
	Help individuals to develop more Calm, Peaceful, and Active appraisal patterns, which has been associated with improved cognitive functioning
**Post-Surgery**	
QOLAP as communication tool	Use the QOLAP tool to support realistic expectations and better coping, that is focusing on the positive, trusting the doctor's input, and reframing negatives into positives by finding a silver lining even to problems
Encouraging patient activation and education	Use motivational coaching, to help patients become more engaged over the recovery trajectory. Coaching low-activation patients could focus on increasing awareness of the different appraisal processes
	Incorporate strategies to increase patients’ level of activation that may improve motivation and adherence to post-surgical rehabilitation. Such motivational coaching could help them become more involved in their healthcare and become more resilient and adherent to physical therapy and home exercise during early recovery from spine surgery. Consider making adherence to PT and home exercise be a secondary outcome
	Treatment may unmask other symptoms so address this in expectations
Promoting adaptive cognitive-appraisal processes	After surgery, help patient to shift to an emphasis on the positive, and to focus on aspects of their life that are more controllable and can reasonably be addressed
	Help patients to focus on the negative, the stable (versus volatile), and solving problems. Acknowledge the volatile and the emotional, but shift the focus to the stable and solveable aspects of their life
	Help to minimize symptom problems so patients can feel better sooner. To do this, patients must focus on big-picture concerns and not on recent episodes. Find a balance between acknowledging what one's challenges are and allowing oneself to appreciate and be grateful for what is working well
	At 3 months post-surgery, encourage patients to expect and focus on symptom relief, and aim for a balanced perspective, thinking about what they can do and have accomplished, rather than the negative or difficult aspects of their recovery trajectory
	Be mindful of frequent comparisons to healthier peers or emphasizing the negative as these appraisal processes are prone to worse outcomes
	As compared to patients who were Stably Well in terms of their depression, comparing oneself to others and emphasizing the negative were associated with worse depression across groups with other trajectories in their depression symptoms. Helping people to shift from such appraisal processes would be a worthwhile focus of cognitive-behavioral therapy
	Address mental health concerns during the spine surgery recovery trajectory Learning to focus on the positive aspects of one’s life and to avoid comparing oneself to others are key to better mental health before and after spine surgery Help patients to shift their focus to emphasize the positive and not recent flare-ups, the future, or their relationships. The clinical team might help them to downplay comparing themselves to others. Over time, patients might be coached to focus much less on the seriousness of their condition or what their doctor told them, and again to find and emphasize the positive in their current experience
	The appraisal processes that were most salient included patterns of emphasis related to getting used to and handling demands or recent changes, problem-solving goals, and comparing oneself to similar others
	The value of maintenance: Help to emphasize that maintaining is a viable and desirable goal even while focusing on solving and accomplishing other matters
	The value of noticing small changes: Focus on the changes in pain, physical and mental health functioning, and notice that as time goes on, one can recognize and be grateful for ever-smaller changes that actually matter
Address mental health	Mental health should be explicitly addressed in all sessions because poor mental health colors the patient's focus after surgery to be more negative
	Help individuals to develop more Calm, Peaceful, and Active appraisal patterns, which has been associated with improved cognitive functioning
	For individuals with long-standing disabling conditions, the intervention might focus particularly on improving their social-support network more than changing appraisal processes. They might seek to create contexts that are more similar to work environments with similar benefits: regular social contact, a meaningful role that is valued, and some kind of reward or compensation for regular and sustained participation
Improve social-support network	For individuals with long-standing disabling, the intervention might focus partituclarly on improving their social-support network more than changing appraisal processes. They might seek to create contexts that are more similar to work environments with similar benefits: regular social contact, a meaningful role that is valued, and some kind of reward or compensation for regular and sustained participation

**Table 2:** Targeted intervention to address cognitive-appraisal processes for specific subpopulations.

Subpopulation	Timing	Topic	Content
Individuals who are low in social support	Pre-surgery	Promoting adaptive cognitive-appraisal processes	Appraisal processes characterized by emphasizing the negative were particularly deleterious
Individuals experiencing greater financial challenges	Pre-surgery	Promoting adaptive cognitive-appraisal processes	Appraisal processes focused on obligations and getting used to changes were protective
Individuals with long-standing disabling conditions	Pre-surgery	Promoting adaptive cognitive-appraisal processes	The intervention might focus particularly on improving their social-support network more than changing appraisal processes. These patients might seek to create contexts that are more similar to work environments with similar benefits: regular social contact, a meaningful role that is valued, and some kind of reward or compensation for regular and sustained participation
	Post-surgery	Address mental health	
		Improve social network	
Individuals taking opioid medication daily before surgery	Pre-surgery	Promoting adaptive cognitive-appraisal processes	This subpopulation is at at high risk of OUD. For these individuals, interventions might emphasize the adoption of more problem-resolution goals at pre-surgery. The lagged effect of baseline goals points to the importance of early intervention
Individuals with low activation	Post-surgery	Encouraging patient activation and education	Focus on increasing awareness of the different appraisal processes
Individuals with persistent or episodic depression	Post-surgery	Promoting adaptive cognitive-appraisal processes	As compared to patients who were Stably Well in terms of their depression, comparing oneself to others and emphasizing the negative were associated with worse depression across groups with other trajectories in their depression symptoms. Helping people to shift from such appraisal processes would be a worthwhile focus of cognitive-behavioral therapy

Both periods also prioritize talking explicitly about the individual’s mental health because low levels of mental health are prominent and long-lasting in people dealing with long-term physical illness and/or disability (25, 28). By addressing depressive symptoms, it is hoped that individuals will acknowledge their experience and be more open to considering appraisal processes associated with improved mental health after spinal surgery.

The pre-surgical intervention sets a stage for engagement and planning their imminent rehabilitation [i.e., “prehabilitation” (33, 39)], directly addressing the individual’s context and their expectations of what they will be able to do and when. Its educational component focuses more on understanding the normal course of recovery after surgery which breaks the recovery period and related functional goals into smaller steps by week since surgery. The appraisal intervention would, at this time, focus attention on encouraging appraisal processes that have been consistently associated with better outcomes across conditions and over time. These processes include, for example, emphasizing the positive, and de-emphasizing how others see oneself or ideal standards of comparison. It would emphasize what their doctor told them would happen, and would help the individual to focus on calm, peaceful, and active ways of thinking, and getting used to changes.

The post-surgical intervention would continue to encourage patient engagement in the recovery process, via Motivational-Interviewing-based coaching to improve adherence to physical therapy, to reduce use of opiates as soon as possible, and to engage in a regular and increasingly challenging home exercise program. This segment would explicitly address the issue of “unmasked” symptoms after successful surgery, i.e., that back pain may be “unmasked, or experienced more saliently” once the leg pain is addressed. This discussion is important for helping patients to recognize the various improvements due to surgery and to adjust their expectations, and thus to increase their emphasis of the positive. Over the course of the initial 3 months since surgery, the intervention would aim to help individuals focus on the positive changes they are experiencing, notice the large and small improvements, to value maintenance of the good aspects of their life and functioning, and to think about the “big picture” of their life rather than small set-backs. These approaches are aimed at helping individuals emulate Symptom Minimizers rather than Symptom Maximizers (31), which is more likely to help them feel better sooner.

This segment also encourages individuals to take steps known to improve their sense of connection to others and social support network, which is mood-enhancing. Given that the individuals have suffered a long-standing and disabling health condition, it is likely that their world contracted, seeing friends less often or fewer friends altogether. This segment would aim to help individuals to identify new environments that they can access that would lead to more regular social interaction and to having a more meaningful role that is valued and would enable sustained participation.

## Discussion

The present work takes hold of the past two decades of cognitive-appraisal research with an eye toward clinical intervention. Our findings suggest an appraisal-assessment based approach to helping individuals adapt better after a large health state change. Building on fundamental applied research on a broad range of patient populations, the appraisal-assessment based intervention seeks to encourage more positive ways of thinking about QOL, and to discourage more negative ways of thinking. These foci are consistent with the intent of CBT interventions, but the QOLAP tool provides a more efficient strategy for elucidating the content of patients’ concerns and how they are thinking about those concerns. It can help patients gain insights into these often unexamined aspects of cognitions, and to facilitate their recognition when their cognitions change. It will be useful to consider four different scenarios illustrating how this appraisal-assessment based approach could be used in the context of spinal surgery.

### Decisional uncertainty

At any point in treatment, patients may be faced with choices that can have short or long-term ramifications for their QOL. In shared decision-making, individuals are asked to weigh pros and cons of different alternatives to help them arrive at choices. However, explanation of the clinical ramifications of different treatments may not be sufficient. It may be challenging for individuals to weigh the implications of different clinical choices for their competing concerns. For example, an individual may be faced with deciding whether or not to delay a surgical procedure. Appraisal assessment here might begin with consideration of personal goals and goal attainment. What is the individual trying to accomplish at present? Can these goals be delayed until after surgery? Would certain priorities have to be abandoned? Another consideration might involve the extent to which the day-to-day experiences of discomfort or disruption of activities overwhelms their current sense of QOL. Is it worth putting up with pain now to avoid interruption of activities to achieve time-dependent personal goals? In the context of shared-decision making, QOL appraisal assessment can provide both patient and provider with the broader context needed to truly evaluate pro’s and con’s with respect to the individuals’ life circumstances.

### Adapting to new diagnosis or worsened prognosis

Patients’ priorities and criteria for QOL can be upended by bad news. Patients in the midst of handling a crisis are not sure whether and how they can maintain valued personal goals, what new problems they need to manage, and what responsibilities they need to abandon. Appraisal assessment can help overwhelmed patients explicate and clarify their different challenges and priorities. This may be an adjunct to Dialectical Behavioral Therapy-based strategies, to help people better understand what they need to accept but also what new possibilities may be open to them. Rather than abandoning certain priorities, assessment of the relative weight that individuals implicitly give to various life domain and concerns (i.e., their combinatory algorithm) may open the door to discussion of reprioritization. Of course, advancing illness also has social implications. Individuals’ standards of comparison for appraising QOL invoke people’s perceptions of how they may be perceived and judged by others. Individuals may also find that they are evaluating themselves and their circumstances based on others’ actual or perceived expectations.

### Symptom management and functional impairment

Even with a positive or stable prognosis, management of symptoms and functional limitations can present a major challenge to surgical patients. One issue is the variability and predictability of symptoms. This dimension is not included in standard patient-reported outcomes assessments. For example, a given level of pain may be referring to a patient’s worst pain or average pain, the experience that was most disruptive or most recent, or pain before or after taking medication. Appraisal assessment can illuminate the timing, frequency, severity and context of self-reported difficulties. Assessment of personal goals also sets the stage for a personalized examination of functional status: How are individuals’ goal-attainment activities affected by functional issues? For example, a patient may really value hosting visitors at home. This may not be something that the patient would spontaneously mention to their surgeon because it does not seem clinically relevant. However, inability to perform routine tasks involved with entertaining may significantly contribute to social isolation and diminished QOL. With greater awareness of these circumstances, clinicians may be able to help patients to optimize their functioning or obtain additional support to maintain a valued activity.

### Establishing patient engagement and satisfaction with care

At its core, appraisal assessment is a way of asking patients “what matters to you”? Incorporating appraisal into patient support and education ensures that care is personalized and responsive. This assessment can be particularly valuable in identifying and overcoming clinicians’ assumptions about patients. Surgeons necessarily hold expectations about patients’ lifestyles and priorities. However, there may be activities and roles important to patients’ QOL that may fall outside the norm. Asking about these issues may lead surgeons to recommend ways patients can better adapt and maintain their activities. Appraisal assessment also opens the door for problem-solving with patients to identify alternative solutions when it is not possible for patients to return to their valued roles. Even if patients need to lower their expectations about surgical outcomes, incorporating appraisal assessment into patient-provider communication may help individuals feel heard and understood, contributing to greater satisfaction in care.

In addition to the general and spine-surgery specific information provided above, Table 2 provides clinicians with further details concerning targeted interventions for specific patient subpopulations, including those who are taking opioid medication daily before surgery and those who have low social support, greater financial challenges, long-standing disabling conditions, low activation, and persistent or episodic depression. For example, clinicians might work with patients taking daily opioid medication to adopt more problem-resolution goals before surgery. As another example, research suggests that individuals with long-standing disabling conditions would benefit from pre-surgery support that promotes adaptive appraisal processes and post-surgery support focused on improving their mental health and expanding their social network. These patients might seek environments that provide regular social contact and meaningful, valued roles (e.g., volunteer position).

### Caveats

The present work focused on clinical applications of the QOLAP_v2_-short form, which is a flexible selection of 84 closed-ended items that were validated over the past two decades of research. While this tool is useful in many situations, there are contexts where the original QOLAP (v1) would be more helpful. This version includes open-ended questions about the meaning of QOL, personal goals, and goal attainment; as well as closed-ended items about Sampling of Experience, Standards of Comparison, and patterns of emphasis (i.e., Combinatory Algorithm). The QOLAP_v1_ is an effective tool for more non-directive and open-ended intervention, such as clinical encounters following the What Matters to You (62, 63) paradigm. Our experience is that this assessment approach helps individuals to reflect upon aspects of their lives that they may not typically consider. People faced with overwhelming life circumstances may have lost touch with more fundamental questions about what QOL means to them. By focusing discussion on what people care about, this assessment may lead to an awareness of how different their life is from their ideal. Such an awareness can trigger change. Despite how painful this may be, individuals have expressed appreciation for the empathic connection created by open discussion of these issues.

The limitations of the present work should be noted. This review is of a specific body of work done largely by members of our group. It is not intended to be a “systematic review,” which would include large numbers of papers by other researchers. While this may seem to limit interpretation of the findings from one perspective, it may also be seen as a strength in that the evidence synthesis was thorough and based on a comprehensive understanding of study findings despite the focused defined scope.

### Real-world implementation and feasibility

The proposed intervention is straightforward, in that it relies on an existing and validated approach for asking about appraisal processes. It thus requires little training or expertise to administer. Interpreting appraisal profiles would entail examining patients’ responses to gain an understanding of their patterns of emphasis and noticing whether patients emphasize cognitive patterns that have been found to be associated with worse outcomes (Tables 1, 2). Potential barriers to adoption and scalability include workflow integration, resource constraints, or variability in clinical familiarity with appraisal concepts. Addressing these potential barriers might be accomplished by providing continuing education programs based on the present work, and focusing the introduction of appraisal-focused conversations to clinical settings that allow more time in the clinical encounter, such as rehabilitation programs or community-based clinics.

### Next Steps

The present work seeks to provide a translational approach toward a new clinical intervention for medically ill patient populations. The next step is clearly to test the proposed intervention in a rigorous clinical trial design. After all, the literature reviewed is generally based on observational cohort study designs, not randomized controlled designs. Accordingly, this current empirical base suffers from a “chicken-and-egg” question: are positive appraisals supportive of better outcomes or are better outcomes causally related to more positive appraisals? (41).

Future research should thus formally test this research question using a randomized controlled trial (RCT). In this RCT, random assignment would be used to place some patients in a control or standard-of-care condition, and some in a cognitive coaching intervention where more positively focused appraisals are emphasized. Then, the impact of the intervention would be compared as a function of improvement in spine-related disability (primary outcome). Secondary outcomes might include adherence, satisfaction, and mental health.

## Conclusion

It is our hope that the present work has provided the next logical step in response-shift research, moving from basic, foundational findings to implications for clinical interventions that can help medically ill patients recover in more lasting and deeper ways from surgery and other health state changes. Our specific experience discussed herein has been on the spine surgery patient population. Further study is needed to determine the generalizability of the results. However, based on response-shift theory (2), we posit that the proposed appraisal-assessment based process can generalize to address the impact and implications of a wide range of catalysts including surgery, a new medical diagnosis, or other life-changing event.
